# Draft Genome Sequence of *Pseudomonas* sp. Strain 10-1B, a Polycyclic Aromatic Hydrocarbon Degrader in Contaminated Soil

**DOI:** 10.1128/genomeA.00325-15

**Published:** 2015-05-07

**Authors:** Maryam Bello-Akinosho, Rasheed Adeleke, Dirk Swanevelder, Mapitsi Thantsha

**Affiliations:** a Agricultural Research Council, Institute for Soil Climate and Water, Pretoria, South Africa; b Agricultural Research Council, Biotechnology Platform, Onderstepoort, South Africa; c Department of Microbiology, University of Pretoria, Pretoria, South Africa; d School of Biological Sciences (Microbiology), North-West University, Potchefstroom Campus, South Africa

## Abstract

*Pseudomonas* sp. strain 10-1B was isolated from artificially polluted soil after selective enrichment. Its draft genome consists of several predicted genes that are involved in the hydroxylation of the aromatic ring, which is the rate-limiting step in the biodegradation of polycyclic aromatic hydrocarbons.

## GENOME ANNOUNCEMENT

Pollution of soil with persistent organic pollutants, especially polycyclic aromatic hydrocarbons (PAHs), is a global problem. Such organic pollutants have been a consequence of various industrial processes that have spared no part of the ecosystem (1). Bioremediation is a promising solution for remediating these pollutants in soils in a cost-effective and environmentally safe manner (2). Bacteria are known to play an active role in degradation and eventual remediation of organic pollutants in soil during this process (3).

Agricultural soil with no history of pollution was collected from the research farm of the Agricultural Research Council, Vegetable and Ornamental Plant Institute, Pretoria, South Africa. The soil is of alluvial origin classified as an Oakleaf soil form (South African classification). It was artificially contaminated in the laboratory with used car engine oil containing a high PAH content. *Pseudomonas* sp. strain 10-1B was isolated from a bacterial community that endured this artificial contamination. *Pseudomonas* spp. are particularly known for their highly versatile metabolic activities in various ecosystems, including soil (4). The strain investigated here was identified as a *Pseudomonas* sp. on the basis of its 16S rRNA gene sequence analyses. *Pseudomonas* sp. strain 10-1B was confirmed to be PAH-degrading through culture characterization after selective enrichment using PAH as the sole carbon source (5), followed by identification and molecular characterization of the Gram-negative PAH-degrading gene in the draft genome presented here.

Genomic DNA of strain 10-1B was isolated using a Macherey-Nagel NucleoSpin soil kit and sequenced on a MiSeq sequencer (Illumina) at the ARC’s Biotechnology Platform, using the Nextera sample preparation kit and the 300-bp paired-end V3 Illumina chemistry. The reads were trimmed on quality, ambiguous bases, and adapters before being merged and *de novo* assembled using CLC Genomics Workbench version 7.5 (CLC bio, USA). Default assembly settings were used but with a maximum word size of 64 bp and contig cutoff of <1 kb. A total of 102 contigs were obtained, with an *N*_50_ of 125,760 nucleotides and a total genome size of 6,346,514 nucleotides, for the draft genome of *Pseudomonas* sp. strain 10-1B. The mean G+C content of the draft genome was 62.5%. The genome was annotated using the NCBI Prokaryotic Genomes Automatic Annotation Pipeline (PGAAP; http://www.ncbi.nlm.nih.gov/genome/annotation_prok). The draft genome annotation predicted 5,584 protein-encoding genes and 5,361 coding sequences (CDSs), in addition to 71 RNAs, of which 65 were tRNAs. In addition, several dioxygenases, reductases, ferredoxin, and Rieske proteins, all known to play an active role in biodegradation processes (6), were identified. Other genes, such as dehydrogenases, identified as being involved in hydrocarbon degradation, were also predicted within the draft genome.

### Nucleotide sequence accession numbers.

This whole-genome shotgun project has been deposited at DDBJ/EMBL/GenBank under the accession number JYKS00000000. The version described in this paper is version JYKS01000000.

## References

[1] Baveye P, Bladon R 1999 Bioavailability of organic xenobiotics in the environment, p 227–248. *In* Baveye P, Block J-C, Goncharuk VV (ed), Bioavailability of organic xenobiotics in the environment. Proceedings of the NATO Advanced Study Institute, NATO Science Partnership Subseries 2, vol. 64. Springer, Dordrecht, Netherlands.

[2] Juwarkar AA, Misra RR, Sharma JK 2014 Recent trends in bioremediation, p 81–100. *In* Parmar N, Singh A (ed), Geomicrobiology and biogeochemistry, Soil Biology 39. Springer, Berlin, Germany.

[3] Hesham AE-L, Mawad AM, Mostafa YM, Shoreit A 2014 Biodegradation ability and catabolic genes of petroleum-degrading *Sphingomonas koreensis* strain ASU-06 isolated from Egyptian oily soil. BioMed Res Int 2014:127674. doi:10.1155/2014/127674. 25177681 PMC4142378

[4] Peix A, Ramírez-Bahena MH, Velázquez E 2009 Historical evolution and current status of the taxonomy of genus *Pseudomonas*. Infect Genet Evol 9:1132–1147. doi:10.1016/j.meegid.2009.08.001. 19712752

[5] Hilyard EJ, Jones-Meehan JM, Spargo BJ, Hill RT 2008 Enrichment, isolation, and phylogenetic identification of polycyclic aromatic hydrocarbon-degrading bacteria from Elizabeth River sediments. Appl Environ Microbiol 74:1176–1182. doi:10.1128/Aem.01518-07. 18156326 PMC2258593

[6] Jouanneau Y, Martin F, Krivobok S, Willison JC 2011 Ringhydroxylating dioxygenases involved in PAH biodegradation: structure, function, biodiversity, p 149–175. *In* Koukkou A-I (ed), Microbial bioremediation of non-metals: current research. Caister Academic Press, Norfolk, United Kingdom.

